# Critical Self-Appraisal Towards the Better Use of a Webinar Series as an Online Tool for Postgraduate Teaching

**DOI:** 10.7759/cureus.20976

**Published:** 2022-01-05

**Authors:** Bharati Mehta, Abhinav Dixit, Om Lata Bhagat, Prasunpriya Nayak, Shival Srivastav, Pooja Ojha, Archana Gaur

**Affiliations:** 1 Physiology, All India Institute of Medical Sciences, Jodhpur, Jodhpur, IND; 2 Physiology, All India Institute of Medical Sciences, Bibinagar, Hyderabad, IND

**Keywords:** online teaching tool, postgraduate teaching, conducting a webinar, webinar on research methodology, webinar planning

## Abstract

Introduction: The current coronavirus disease 2019 (COVID-19) pandemic adversely affected the conventional teaching mode, resulting in an exponential rise in online modalities such as webinars. Simultaneously, the lockdown provided substantial time to pursue potential academic content on the web. It is known that newly admitted postgraduate students of Physiology require a structured program that can guide them to conduct research for the completion of the course.

Methods: Gauging the opportunity, a webinar series was conducted on basic research methodology and thesis writing in Physiology. The series comprised hourly lectures delivered between 4:00 and 5:00 pm for seven consecutive days. Suggestions for future topics for webinars were sought through open-ended questions. Additionally, feedback for increment in students' knowledge at the end of the webinar was also inquired on a Likert scale. Open-ended answers were pooled into fields, and Likert scale scores were evaluated.

Results: There were 364 (35.8%) postgraduate students who registered for the webinar. The remaining were faculty (51.6%), research scholars (8.8%), and senior residents (3.8%). Among the postgraduate students who submitted the feedback, a majority (98.4%) of them agreed that their knowledge was enhanced at the end of the series. Most of the postgraduate students (31%) chose Biostatistics for future webinars.

Conclusion: Webinars are a useful tool for postgraduate teaching. They should be constructed with engaging infrastructure and relevant examples. The availability of recorded content on the online forum is beneficial for asynchronous learners. Having an idea about students’ choice for essential topics helps in the advanced planning of a demanding webinar.

## Introduction

The restrictions imposed during the current coronavirus disease 2019 (COVID-19) pandemic have led to an unprecedented change in teaching modalities, making it more of an online process [1-2]. Video conferencing, webinars [3], and now e-conferences have shown potential utility in exchanging thoughts and continuing learning in these difficult times. Despite the advantages of geographical flexibility and feasibility for more participants in a webinar [4], holding the attention of participants on a virtual platform is challenging. Seemingly, designing an effective online event requires meticulous planning and preparedness for troubleshooting, besides presenting a strong theme in an exciting manner [5].

Quest for a suitable theme for the present study encountered the fact that choosing a thesis topic, conducting quality research, and publishing the same are fundamental challenges for a postgraduate student taking admission in physiology. An assessment of awareness regarding research among postgraduate students has shown that despite a positive attitude towards research, there is a need to implement a conducive research environment among resident doctors [6]. Notably, most institutes do not have an established research orientation program for postgraduate students. Hence, gauging the rise of online teaching trends and the concurrent need for research and subsequent publication by the budding physiologists [7], this relatively important theme was chosen to be addressed through a web-based lecture series akin to a virtual workshop during COVID-19.

Feedback is an essential step towards more rational planning in the future and matching the learner's expectations [8]. It communicates participants' receptiveness to the teaching tool and the particular theme [9]. Thereby, the present study also obtained students' feedback for the webinar-based program, which imparted knowledge about the research methodology and publication in physiology through an online questionnaire-based approach.

## Materials and methods

Ethical permission was obtained from the Institutional Ethics Committee. Participants' informed consent was sought through an internet-based questionnaire tool before the inclusion of their data for analysis.

Logistics and planning

**Figure 1:**
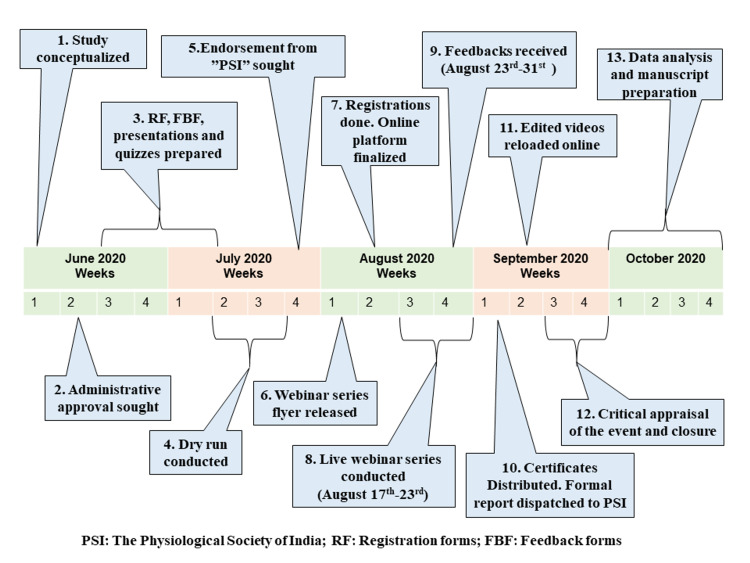
Event chronology

Faculty members, technical experts from the information technology section, and senior advisors from the same institute comprised the team. Planning was focused on preparing a customized program for the target audience, identified as postgraduate students and research scholars. The existing infrastructure, the host institute's network, and freely available online platforms were planned to be used. The resource faculties were pleased to contribute academically without any honorarium. The team members designed a webinar checklist to keep track of minute details. Event chronology has been presented in Figure 1.

Program schedule

The webinar series was centered on "Basic Research Methodology and Thesis Writing in Physiology." Relevant lecture topics, speakers, and live streaming timing were finalized after several discussions. Details of topics and speakers can be accessed from the online link provided in the video editing, introspection, and closure section.

The faculties taking lectures were from the same department of the institute. Considering that a whole-day workshop can be tiring, the team decided that webinars of an hour-long duration should be delivered in a series spanning over seven days to complete the entire content. Understanding a concept is increased by giving examples; hence, many examples from a physiology research perspective were included. A standard PowerPoint (Microsoft Corporation) background, bearing the Physiological Society of India (PSI) logo and our institute logo, was designed to depict a common webinar theme and align connectivity between the lectures.

Dry run

An online run of the whole event was conducted. The postgraduates of our department attended the dry run, and their suggestions were also incorporated. Self-appraisal after the dry run is presented in Table 1.

**Table 1:** Pre-program self-appraisal

Serial number	Points considered
1	Check the suitability of the online platform for the accommodation of the participants
2	Identify online streaming-related technical glitch
3	Check content and continuity between lectures
4	Check structural details of the presentation
5	Maintain time management for each session
6	Identify the precise role of each team member, familiarize with the protocol, and enable troubleshooting

Physiological Society of India (PSI) platform

The success of the dry run motivated the team to expand the catchment on a national level. Hence, for maximum reach to the postgraduate students from different regions of the country, "The Physiological Society of India (PSI)" (a national-level body) was requested for collaboration, and their response was affirmative.

Advertisements and registrations

Program flyers were released on several Internet-based message service groups 10 days before the commencement of the event. The program schedule and the link to the Internet-based questionnaire tool for registration were provided with the flyer. Registration to attend the event was free of cost but mandatory. Each participant was provided with a unique registration number at the time of registration. An Internet-based message service group was made for the participants finding it difficult to register.

Upgradation of online platform and creation of Internet-based video streaming channel

The initial estimate of participants was 100 participants, but as registrations poured in, the numbers hit a thousand, calling for upgrading the online platform to accommodate all the participants. Hence, live streaming on an Internet-based video streaming platform, specific for large group accommodation, was contemplated. Finally, a specific live streaming platform was selected considering its' technical simplicity and financial aspects.

An Internet-based video streaming platform channel was created for the event, and emails were sent to all the registered participants, sharing the channel's link. Textual and pictorial directions for subscribing to the channel and joining the event were provided. The team promptly addressed any technical difficulty. Upon clicking the channel, participants were greeted with a welcoming slide show. It included details of the topics, speakers, program schedule, and a "see you soon" message.

Live streaming sessions from 3:45-5:00 pm

All sessions began with a welcome message from the Director of the institute, dignitaries from PSI, and the organizing chairman, and some ground rules were stated, all woven into a slide show with soft background music. This slide show continued for 15 minutes till the clock struck 4:00, and the session began. Few initial glitches were identified and tackled, enabling smoother streaming subsequently. The chatbox was continuously monitored for any question or suggestion. Individual queries were addressed at the end of the session by the speaker through emails, and suggestions were acknowledged. During streaming, messages and calls from the students joining late were received, requesting to share the content they had missed. It was informed that besides live streaming, videos will be available online and can be accessed at their convenience. Every session culminated with a few important notes regarding the submission of feedback forms and issuance of certificates.

MCQ-based self-evaluation

Each lecture was followed by a multiple-choice-question-based self-assessment having five questions that were presented through an internet-based questionnaire tool. The link to the same was active for one day till the next session started. The questions were in line with the intended specific learning objectives for that session. Questions framed by the speaker (weighted by the team) were piloted in the in-house postgraduate students during the dry run. Answers and explanations to the questions were made available immediately upon submission of the answers to all questions. The purpose of providing MCQs to the postgraduate students was to let them assess their understanding of some important topics from the lecture.

Feedback from participants

Whereas the self-assessment quizzes were not mandatory, the submission of feedback was compulsory for obtaining the participation certificate. The last lecture was followed by a link to submit the feedback form, which included the consent to include participants' responses in the study. Suggestions and open-ended views on future topics were obtained from the participants. Students' perception of the utility of individual sessions in skill enhancement was additionally inquired using the Likert scale. Among six points, '0' was no improvement and '5' was extremely improved. The overall improvement in knowledge and the utility of the program were assessed using a five-point Likert scale. 'Strongly disagree' was marked '1' and 'Strongly agree' with '5'. Certificates relevant from an academic viewpoint were issued to all participants submitting their feedback.

Video editing, introspection, and closure

Live-streamed videos were edited for quality and reloaded online on the same channel after completing the webinar series. Videos are still accessible from the given link https://www.youtube.com/channel/UCP5FdtPYyR7ndXAPbKaAdjQ/videos.

In a final meeting of the team members, program appraisal and critical analysis were done again, and points were noted for future events. Certificates were provided to the resource faculties.

The number of registrations, feedback, chat interactions, and postgraduate students' satisfaction with the event were assessed. Answers to open-ended questions were analyzed for the core idea and subsequently pooled into fields. The students' concordance in skill enhancement with each session and motivation to take up MCQs was also assessed.

## Results

**Figure 2:**
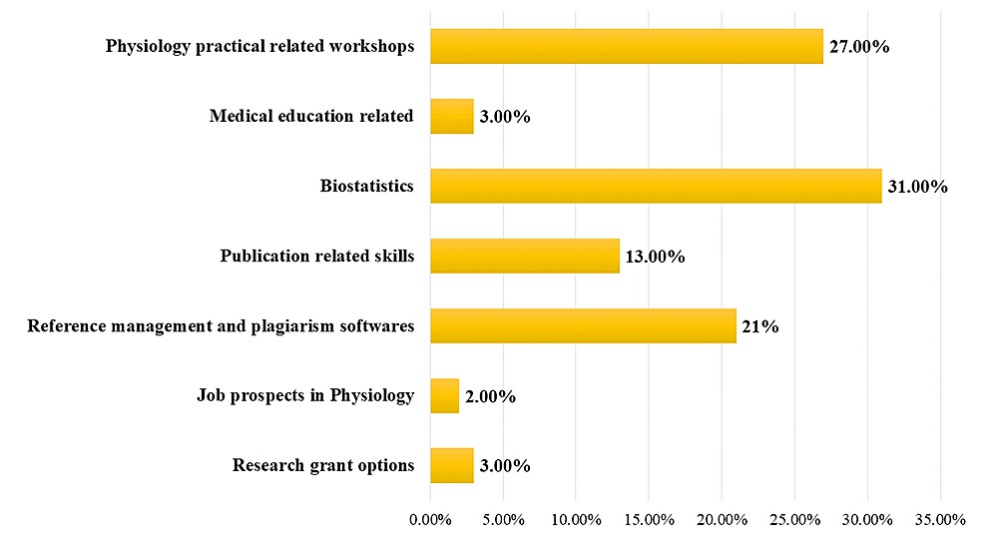
Postgraduate students’ choices for future webinar topics

One-thousand seventeen registrations were made for the event from different states of the country. There were 364 (35.8%) registrations by the postgraduate students. The remaining participants comprised 90 (8.8%) research scholars, 525 (51.6%) faculty, and 38 (3.8%) senior residents. At the end of the entire event, 394 participants submitted the feedback form, and among them, 364 provided consent to include their responses in the study (participation for this study was 92.4%.). Out of those who gave consent, there were 187 postgraduate students. The postgraduate students’ responses to open-ended questions seeking suggestions for the future topic are presented in Figure 2.

Postgraduate students’ response to webinar sessions and their performance in the assessment test is presented in Table 2.

**Table 2:** Postgraduate (PG) students’ response to sessions in feedback, and self-assessment

	Feedback (Scores 4, 5)		Self-assessment		
	All participants	PG students	PG students/ All participants attempting MCQ (%)	Scores >60%	
	n=364	n=187		All participants	PG Students
Session 1	79.9	78.6	137/246 (55.6)	230 (93.4)	120 (87.6)
Session 2	87.3	82.3	112/192 (58.3)	184 (95.8)	100 (89.2)
Session 3	84.0	80.2	102/199 (51.2)	133 (66.8)	66 (64.7)
Session 4	79.4	75.9	91/184 (49.5)	120 (65.2)	58 (63.7)
Session 5	71.9	70.1	82/166 (49.4)	134 (80.7)	58 (70.7)
Session 6	79.1	78.0	69/148 (46.6)	92 (62.1)	44 (63.7)
Session 7	83.5	80.2	79/160 (49.3)	147 (91.8)	69 (87.3)
Average of all sessions	80.7	77.9	96/185 (51.8)	148 (80.0)	73 (76.0)
The feedback column shows the percentage of participants who selected scores 4 and 5 on a 6-point Likert scale, wherein '0' was no improvement, and '5' was extremely improved. Data presented in the self-assessment columns are the number of participants. The numbers expressed in parenthesis are percentages.					

The majority of postgraduate students (98.4%) agreed to an overall increase in knowledge after the webinar. A report was submitted to the PSI after the culmination of the entire event.

## Discussion

The current webinar series was conducted to deliver research methodology content to the postgraduate students through a webinar series over seven days. Though the program theme was primarily targeted towards the benefit of postgraduate students and research scholars, the participation of faculty and senior residents (563; 55.4 %) clearly indicated that the webinar theme was very apt and attractive to the Physiology community. The webinar series was engaging, and the submission of feedback was easy. The questions framed in the feedback form were such that the postgraduate students had to compare their knowledge at the end of the session with their pre-existing knowledge, minimizing the invested time (see Appendices). Out of 187 postgraduate students, who gave their consent to include their response in the study, 98.4% agreed to an overall increment in knowledge. Analyzing the average scores of the postgraduate students for all sessions, more than 50% (96) of them were motivated enough to take up self-assessment tests offered at the end of each session, and approximately 76% (73) of those who attempted the questions, got more than 60% of the items correct. Postgraduate students were satisfied with the content and expressed a desire to attend more such webinars.

Webinar evaluation generally includes the number of registrations, response rates, number of responses to the post-webinar survey, and participants' perception of the webinar [10-11]. Since the flyers were shared with the Internet-based message service groups, snowballing may have led to an over advertisement resulting in nationwide registrations from the Physiologists and researchers from other disciplines, senior residents, and faculty members (even professors). It re-emphasizes the relevance of the webinar topic, offered in collaboration with a national level Physiology society besides free registrations. 

Despite the on-air timing for the webinar being 16:00 hours, many students were online 15-20 minutes before the event time, portraying palpable enthusiasm for the series. The number of postgraduate students attending live sessions was maximum on the first day and gradually plateaued over the week. Probably they learned that the videos would remain online for later viewing and watched them at a convenient time. The number of views for each session by the end of next week (more than a thousand) confirmed our claim. To stick to a schedule is often a limiting factor for the webinars and some participants choose to study at their own pace [12]. Webinars are a prototypal form of synchronous learning. Still, in the scenario of accessibility to all videos for online viewing, it can be regarded as a blend of synchronous and asynchronous learning, catering to both types of audiences.

The duration and frequency of webinars have been found to affect its success and form an integral part of webinar planning rules [13]. Generally noticed, a single session or short webinars are absolutely complacent for the audience; conversely, lengthy lectures demand engaging infrastructure [14]. In the above context, despite being a week-long program, this web series received a welcoming response. Delivering priority content crisply and punctually, valuing participants' needs, time, and efforts were the main points.

While planning an online event, it is advised to match participants' expectations with the program's technical attributes [15]. A post-webinar survey demonstrated the utility of this event for the attendees. None of them opted for "no improvement" in the overall assessment; instead, live chat was flooded with encouraging comments. Besides live chat, the faculties received many laudatory messages on Internet-based message service groups, personal numbers, and emails. Some of the Professors watched the live sessions with their postgraduate students while maintaining social distancing and shared the picture with one of the webinar team members. A few others projected the live broadcast of the webinar on a bigger screen and made it available for undergraduate students as well, sensitizing them towards research methodology. 

Postgraduate students desired to attend more webinars with pertinent research content, and contrary to the expectation, some wanted longer sessions. In agreement with our evaluation, several studies examined the usefulness of online teaching methods during the COVID-19 outbreak. They found it a valuable and reliable modality, though the content delivered by them varied [16-17].

While organizing this event, several hurdles were faced, which led to improvisations and troubleshooting by the team. The lessons learned during the planning, delivery, and assessment of the webinar have been presented in Table 3.

**Table 3:** Post-program self-appraisal

Issues	How we managed it?	Lessons learned for future webinars
Team	The versatile team was very supportive.	Team members should be efficient to improvise at the time of need.
Funding and Infrastructure issues	We used a freely available broadcasting platform. No incidental expenses were incurred during the program. Hence, no funding was required.	The freely available online platforms should be used if a subscribed platform is not available in the institute or if it does not support live and offline streaming. Contingency funds may be helpful at times.
Who to get registered for the event?	The target audience was identified.	A pilot run of the registration form will be helpful to get an idea about which information is required. Questions like “How did you come to know about the event?” or “What is your motivation to attend the event?” will be helpful.
Timing of the webinar	We arranged an offline video lecture for those who could not join the live sessions	The event should be planned in a way to ensure offline availability of sessions after the online event to maximize outreach
Number of participants	Due to increased registrations, we changed the online platform to accommodate a large number of participants.	The expected number of participants should be estimated. The online platform should be able to accommodate all.
Dry run	A prior dry run helped identify the role of each member, familiarize with the protocol, and enable troubleshooting	One should be prepared for the unexpected. Hence, a dry run will be helpful before conducting a webinar.
Sending emails to all participants simultaneously	Emails were sent to the participants through multiple email ids, as it was not possible to send emails to all participants through a single dedicated email (created for the event)	Facility for sending bulk emails should be available
Publication	Accreditation was sought from the Physiological Society of India. The project proposal was submitted to the Institute Research cell and ethical clearance was obtained in advance.	If data obtained from the event is planned to be published, due approval should be obtained from Institutional bodies/Regulatory authorities well in advance.
Assessment	A pilot run of the multiple-choice questions was performed in-house with the departmental post-graduate students.	A psychometric analysis to validate the MCQs would be helpful.
Evaluation	When planning webinar evaluation	Participants’ learning can be assessed effectively through a well-planned pretest before and a post-test after the sessions.
Feedback	A link to the feedback was provided at the end of the last session.	Feedback should include specific challenges faced during the event and satisfaction with the content and its presentation. Open-ended responses may help in getting a general opinion about the event.

The strengths of the present study are that it was a timely organized event, utilizing lockdown and quarantine hours for knowledge enhancement by young researchers seeking current concepts in research methodology. The technical quality of the event was sound, and the punctuality of live streaming was appreciated by the participants. Examples pertinent to research in physiology were beneficial for the postgraduate students, and the live chat was engaging.

The limitations of the present study were that a live question-answer session was not held. This was due to the choice of the Internet-based live streaming platform, which could accommodate a high number of participants but could not provide voice chat options.

## Conclusions

We conclude that webinars are a useful online modality for delivering research methodology content to postgraduate students with adequate satisfaction. Besides live sessions, the availability of online recorded material enhances participants' reach out, as they watch it comfortably at their own pace. A successful webinar on research methodology in physiology needs to be well-planned and efficiently executed besides being engaging and rice with content. Obtaining postgraduate students’ views on future topics for webinars will help address the need of webinars on specific topics.

## Appendices

**Table 4:** Question used for obtaining feedback from the participants

Feedback regarding skill enhancement	
After attending our webinar series, how have your skills improved?	
Rate on a scale of 0 to 5, where 0 means no improvement and 5 means extremely improved	
Q1	Ability to conceptualize a research idea and frame an appropriate title
	5: Extremely improved, 4, 3, 2, 1, 0: No improvement
Q2	Ability to search literature from scientific database and use tools to manage the references
	5: Extremely improved, 4, 3, 2, 1, 0: No improvement
Q3	Ability to design your study appropriately to conduct research
	5: Extremely improved, 4, 3, 2, 1, 0: No improvement
Q4	Ability to frame relevant objectives to pursue your study
	5: Extremely improved, 4, 3, 2, 1, 0: No improvement
Q5	Ability to apply basic statistical methods on your data and interpret results
	5: Extremely improved, 4, 3, 2, 1, 0: No improvement
Q6	Ability to present your data using relevant graphs and tables
	5: Extremely improved, 4, 3, 2, 1, 0: No improvement
Q7	Awareness of ethical policies and guideline to conduct your research
	5: Extremely improved, 4, 3, 2, 1, 0: No improvement
Q8	Ability to compile your results, discuss them and write your thesis/ dissertation and paper
	5: Extremely improved, 4, 3, 2, 1, 0: No improvement
Overall feedback	
Q1	The content of webinar series was useful for me and has increased my knowledge
	Strongly disagree, Disagree, Neutral, Agree, Strongly agree
Q2	Please provide feedback regarding how we may improve the program
Q3	Please suggest areas that we may cover in future webinars to make the content more useful
